# In vitro and in vivo muscle mass and strength during the first week of critical illness

**DOI:** 10.1186/s40635-025-00755-7

**Published:** 2025-06-03

**Authors:** Wout J. Claassen, Isabel M. van Ruijven, Marloes van den Berg, Rianne J. Baelde, Alexcia Fortes Monteiro, Rajvi M. N. Balesar, Sylvia W. Hania, Donald L. van der Peet, Peter J. M. Weijs, Coen A. C. Ottenheijm, Sandra N. Stapel

**Affiliations:** 1 Department of Physiology, Amsterdam Cardiovascular Sciences, Amsterdam University Medical Center, Vrije Universiteit, Amsterdam, The Netherlands; 2 Department of Adult Intensive Care Medicine, Amsterdam Cardiovascular Sciences, Amsterdam University Medical Center, Vrije Universiteit, P.O. Box 7057, 1007 MB Amsterdam, The Netherlands; 3 Faculty of Sports and Nutrition, Center of Expertise Urban Vitality, Amsterdam University of Applied Sciences, Amsterdam, The Netherlands; 4 Department of Nutrition & Dietetics, Amsterdam Movement Sciences, Amsterdam University Medical Center, Vrije Universiteit, Amsterdam, The Netherlands; 5 Department of Physiotherapy, Amsterdam University Medical Center, Vrije Universiteit, Amsterdam, The Netherlands; 6 Department of Surgery, Amsterdam Gastroenterology Endocrinology Metabolism, Amsterdam University Medical Center, Vrije Universiteit, Amsterdam, The Netherlands

**Keywords:** ICU, Muscle mass, Muscle strength

## Abstract

**Background:**

Loss of muscle mass and strength is provoked by critical illness. Our primary aim was to study the development of muscle atrophy and weakness in vitro in isolated myofibers and in vivo muscle mass and in vitro muscle strength during the first week of critical illness. Furthermore, we explored how in vitro muscle strength compares to healthy controls. Finally, we studied correlations between in vitro muscle mass and strength and in vivo muscle mass in critically ill patients.

**Methods:**

We performed a secondary analysis using data from a randomized controlled trial. We studied contractile force of single myofibers isolated from muscle biopsies around admission (day 1–3) and around 1 week after inclusion (day 8–10). Furthermore, we studied myofiber cross-sectional area (CSA), proportion of fast-twitch myofibers, bio-electrical impedance analysis-derived fat-free mass index (FFMI), ultrasound-derived quadriceps muscle layer thickness (QMLT) and diaphragm thickness. In the control group, only contractile force outcomes were available.

**Results:**

In total, ten ICU patients had two muscle biopsies taken. Maximum force of both fast and slow-twitch myofibers was reduced at day 8–10 compared to day 1–3, even though there were no differences in normalized force and calcium sensitivity. FFM and QMLT did not change over time, nor were there differences between groups. Compared to healthy controls, maximum force of myofibers was lower in the ICU group at day 8–10 in both slow and fast-twitch myofibers, while the calcium sensitivity of force was lower in slow-twitch myofibers. We found a significant correlation between myofiber CSA vs. FFMI (*r* = 0.68) and maximum force of the fast-twitch fibers vs. QMLT (*r* = 0.72).

**Conclusions:**

During the first week of critical illness, maximum force declined over time, while no other in vitro parameters changed. We found a moderate correlation between myofiber CSA vs. FFMI and maximum force of the fast-twitch fibers vs. QMLT.

**Graphical Abstract:**

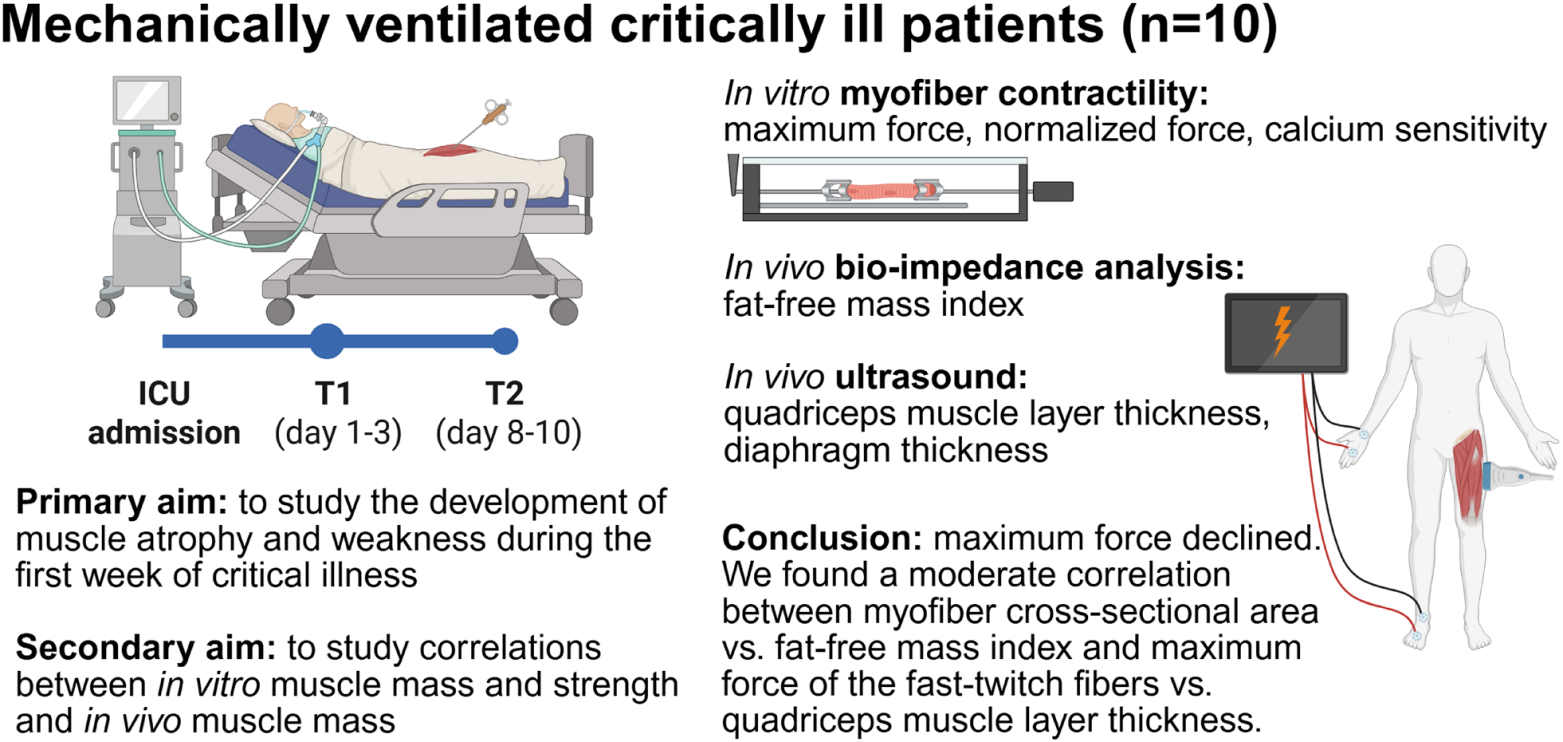

**Supplementary Information:**

The online version contains supplementary material available at 10.1186/s40635-025-00755-7.

## Background

Critically ill patients lose skeletal muscle mass at a rate of up to 2% per day during the first week of the Intensive Care Unit (ICU) admission [1]. Up to 50% of patients develop ICU-acquired weakness (ICU-AW), leading to impaired functional ability and quality of life [1, 2]. We previously showed that low muscle mass at ICU admission is associated with impaired survival and with increased discharge to a nursing home instead of home [3, 4]. Muscle atrophy and dysfunction are induced by systemic inflammation, increased catabolism, immobilization and suboptimal nutritional intake [4].

Muscle mass may be assessed through various modalities, including computed tomography (CT). However, CT-scanning is time-consuming, expensive, involves risks from radiation exposure and patient transportation and scans are not routinely made. Non-invasive, affordable and time-efficient modalities for the assessment of muscle mass in critically ill patients are needed. Bio-electrical impedance analysis (BIA) and ultrasound measurements are currently used [5]. BIA is quick and easy to perform [6]. However, standardized fluid ratios of intra- and extracellular water that are assumed in BIA are often unrealistic in critically ill patients, complicating the interpretation of results in these patients [6]. Ultrasound measurements require more training but may be a useful tool for monitoring muscle mass in the critically ill [7, 8]. Quadriceps muscle layer thickness (QMLT) is often assessed as a marker of muscle mass. Additionally, ultrasound measurement of diaphragm thickness is of interest as diaphragm weakness is common in patients with ICU-AW, and atrophy of this muscle appears to precede loss of peripheral skeletal muscle [9–11].

Besides muscle mass, muscle strength is of great importance, as it determines function. Myofiber contractility is an important factor determining muscle strength [12]. Muscle weakness can result from critical-illness neuropathy, impaired function of the neuromuscular junction or myopathic changes within myofibers [13]. By assessing the contractility of myofibers isolated from muscle biopsies, the function of the sarcomeres within myofibers can be studied. Changes in sarcomere function in limb muscle may contribute to weakness in critically ill patients, as a decrease in normalized force (i.e., force normalized to myofiber cross-sectional area (CSA)) was observed in tibialis anterior biopsies of neuro-ICU patients [14]. Numerous small-molecule drugs that increase muscle contractile force by acting on the sarcomere are under development [15]. If sarcomeric dysfunction underlies limb muscle weakness of ICU patients, these compounds may be used to treat ICU-acquired weakness in the future. Muscle strength eventually determines functional capacity and quality of life in ICU survivors, but studies on contractile performance of skeletal muscle in the critically ill are lacking. Also, monitoring contractility of myofibers is not feasible in daily practice as obtaining muscle biopsies is invasive. To our best knowledge, correlations between in vitro and in vivo assessed muscle mass and strength have not been previously investigated.

Our primary aim was to study the development of muscle atrophy and weakness in vitro in isolated myofibers and in vivo muscle mass and in vitro muscle strength during the first week of critical illness. To account for changes in contractility during the first 1–3 days, we also compared our data to healthy controls. Finally, we studied correlations between in vitro muscle mass and strength and in vivo muscle mass in critically ill patients.

## Methods

We performed a secondary analysis using data from a single-blinded, randomized controlled trial (RCT), which was discontinued due to low inclusion rates. The protocol has been filed on 27 July 2017 in the Clinical Trial Register under #NCT03231540 and was approved by the Medical Ethical Committee of VU Medical Center, Amsterdam, The Netherlands. Informed consent was obtained from the patient or a legal representative.

In this RCT, next to standardized exercise, both groups received enteral nutrition with a protein provision of 1.0 g/kg/day. The intervention group had a protein provision target of 1.5 g/kg/day, but this target was not reached, resulting in a similar protein provision in both groups. An overview of the original study interventions and procedures are presented in Supplementary File.

### Eligibility criteria for critically ill patients

Patients ≥ 18 years that had an expected ventilation duration of ≥ 72 h, were expected to tolerate enteral nutrition ≥ 72 h and had a Sequential Organ Failure Assessment (SOFA) score ≥ 6, were considered eligible for inclusion. Exclusion criteria were contra-indications to enteral nutrition, short bowel syndrome, type C liver cirrhosis or acute liver failure, dependency on renal replacement therapy, requiring other specific enteral nutrition formula for medical reasons, body mass index (BMI) > 35 kg/m^2^, extensive treatment limitations, disseminated malignancy, hematological malignancy, primary neuromuscular pathology, chronic use of corticosteroids for > 7 days before ICU admission or contra-indications for muscle biopsy such as need for continuous systemic anticoagulation, prothrombin time > 1.3 or thrombocytes < 100 × 10^9^/L.

### Eligibility criteria for healthy controls

Healthy subjects ≥ 18 years that had no history of muscle disease were included.

### Outcomes

The primary outcomes were: changes in skeletal muscle strength as measured by contractile force of single myofibers isolated from the biopsies around day 1–3 and day 8–10 after ICU admission: maximum force (mN), maximum normalized force (mN/mm^2^) and calcium sensitivity of force (EC50, [Ca^2+^]) of both slow-twitch and fast-twitch myofibers, muscle mass between day 1–3 and day 8–10 after ICU admission as measured in vitro: by myofiber CSA (μm^2^) and percentage of fast-twitch fibers (%) from cryosections. Secondary outcomes were muscle mass around day 1–3 and day 8–10 after ICU admission as measured in vivo: fat-free mass (FFM, kg) and fat-free mass index (FFMI, kg/m^2^) as measured by BIA and quadriceps muscle layer thickness (QMLT, cm) and diaphragm thickness as measured by ultrasound.

Outcomes for healthy controls were skeletal muscle strength as measured by contractile force of single myofibers isolated from the biopsies: maximum force (mN), normalized force (mN/mm^2^) and calcium sensitivity ((EC50, [Ca^2+^]) of both slow-twitch and fast-twitch myofibers.

### Muscle biopsies

Vastus lateralis muscle specimens were obtained at day 1–3 and day 8–10 after ICU admission, from the ipsilateral leg. Biopsies were stored at − 80 °C. We used a previously described method with minor adaptations for muscle specimen handling [16], which was also used for the controls. We studied the contractile force and the calcium sensitivity of force of myofibers permeabilized with triton-x by activating them with solutions containing incremental concentrations of exogenous Ca^2+^. Within the control and the intervention group, we evaluated whether myofiber contractility changed over time. Slow- and fast-twitch myofibers were analyzed separately because of their distinct contractile properties. A more detailed overview of biopsy analysis, including myofiber contractility, is described in the Supplementary File.

### In vivo measurements of muscle mass

Measurements were performed by a trained member of the study team. BIA and ultrasound were measured on the same day muscle biopsies were taken. BIA was measured using the BIA 101 Anniversary edition device (GLNP Life Sciences, Akern) according to a standardized operating procedure on the ipsilateral side of the body. FFM and FFMI were calculated according to Kyle et al. [17]. Ultrasound measurements of QMLT (ipsilateral side) were taken at 1/3 upwards from the superior margin of the patella to the anterior superior iliac spine using minimal pressure. Diaphragm thickness was measured at the end of expiration.

### Statistical analysis

Given that this is a secondary analysis of a RCT, we did not perform a sample size calculation. Descriptive statistics were presented as mean and standard deviation or median and 25–75th percentile, depending on distribution. Differences between outcome parameters were studied using paired t tests or Wilcoxon Signed-Rank tests, depending on distribution of the data. Differences between the ICU and control group were analyzed using linear mixed models with patients as the random factor. Correlations between in vitro measurements [CSA, maximum force, normalized force and calcium sensitivity (for both slow- and fast-twitch myofibers)] and in vivo measurements (FFMI, QMLT and diaphragm thickness) were calculated. In case of normally distributed data, Pearson’s Rho correlation coefficient was used. In case of non-normally distributed data, Spearman Rho correlation coefficient was used. We used a two-sided significance level of 5% for all analyses.

## Results

### Patients

Paired biopsies were obtained in a total of 10 patients. Individual patient characteristics are presented in Table 1. Most patients were male (90.0%) and admitted to the ICU after traumatic injury (70%). The median BMI of patients was 25.3 [24.5–29.5] and median SOFA score was 10 [6.0–11.3]. Outcome parameters for in vitro and in vivo measurements are presented in Supplementary File, Table S3. The majority of patients had a low (0–1) mobility level during the study period. Characteristics of the healthy controls versus ICU patients are presented in Table 2. Table S4 details the duration of mechanical ventilation at the time both biopsies were acquired. The study day at which the biopsy was performed is presented in Table S5.

**Table 1:** Patient characteristics

Patient	Age (years)	Sex	BMI (kg/m^2^)	Reason for ICU admission	SOFA score	ICU mortality	Mobility level
1	57	M	25.7	Traumatic brain injury	10	No	0–1
2	81	M	24.8	Traumatic brain injury	6	Yes	0–1
3	30	F	27.4	Traumatic brain injury	18	No	0–1
4	84	M	23.2	Traumatic brain injury complicated by meningitis, pneumonia	6	No	0–1
5	24	M	23.9	Traumatic brain injury	4	No	0–1
6	88	M	28.0	Respiratory failure after lobectomy (malignancy)	8	Yes	0–1
7	65	M	34.5	Severe trauma	11	No	0–1
8	60	M	24.7	Severe trauma	10	No	0–4
9	71	M	29.4	Traumatic brain injury	10	No	0–3
10	66	M	29.6	Respiratory failure due to pneumonia	12	No	0–5

**Table 2:** Characteristics of healthy controls and ICU patients

	Healthy controls (*n* = 7)	ICU patients (*n* = 10)	*p* value
Age (year), median [25-75th percentile]	53 [50–58]	63 [32–82]	0.58
Sex (male), *n* (%)	5 (71)	9 (90)	0.32
BMI (kg/m^2^) [25-75th percentile]	26 [23–32]	25 [23–28]	0.36

### Cryosections

**Fig. 1:**
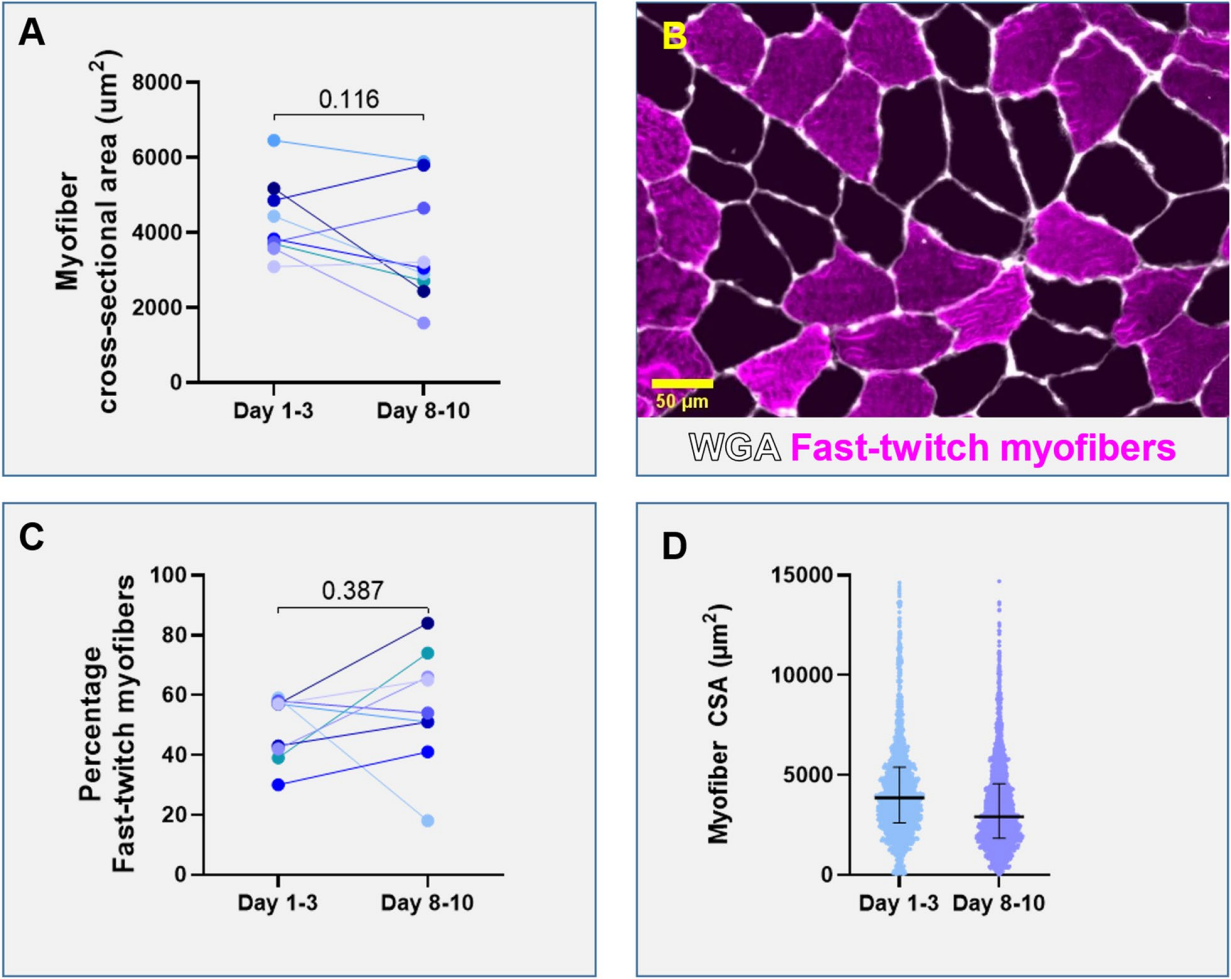
Myofiber cross-sectional area and myofiber type. **A** The cross-sectional area of myofibers of ICU patients at day 1–3 and day 8–10. Data are presented as mean myofiber cross-sectional area per patient. **B** Representative image of the cross-sectional area of myofibers in a quadriceps muscle cryosection (violet: fast-twitch myofibers, black: slow-twitch myofibers, white: wheat germ agglutinin staining of the extracellular matrix). **C** Percentage of fast-twitch myofibers. One symbol represents one patient. **D** Distribution of myofiber cross-sectional area at day 1–3 and day 8–10. One symbol represents one myofiber

Cryosections were cut from the biopsies to study myofiber CSA and type. Table S6 details the number of measured myofibers per biopsy. The myofiber CSA did not change over time (− 737 ± 1254 μm^2^, Fig. 1A), and there were no significant changes in myofiber type (Fig. 1C). Figure 1D shows the distribution of the myofiber CSA.

### Contractile force measurements of single myofibers

**Fig. 2:**
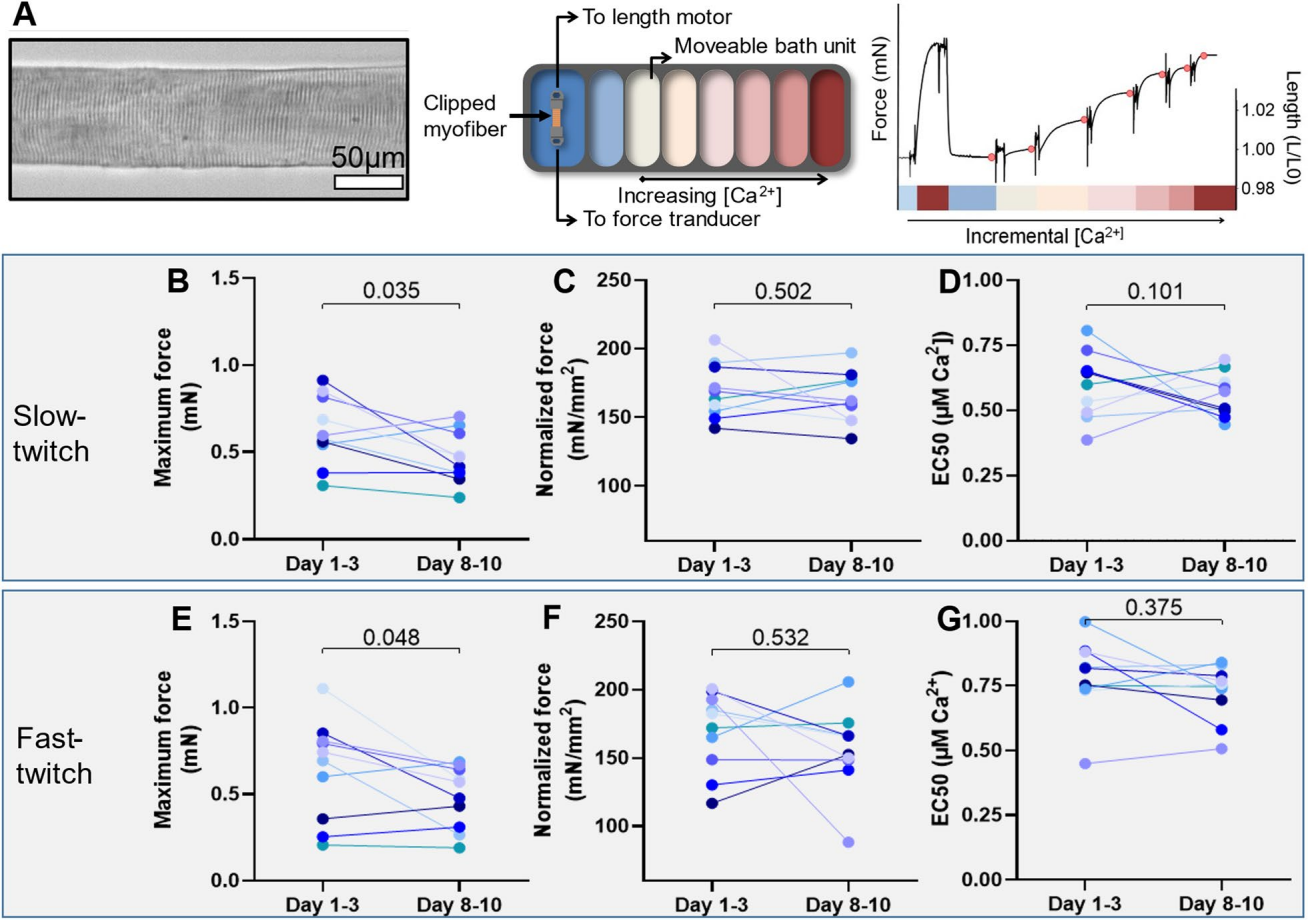
Contractility of single myofibers over time. **A** Example of a diaphragm myofiber while mounted in the experimental setup for contractility assays. Schematic of the experimental setup, which is on top of an inverted microscope, displaying the baths with incremental Ca^2+ ^ concentrations. Example of a force trace during the experimental protocol. Red dots indicate the force values used for analyses. **B** The maximum contractile force generated by both slow- and fast-twitch (**E**) myofibers over time. Normalized force (force divided by myofiber cross-sectional area) over time in both slow (**C**) and fast-twitch (**F**) myofibers. The calcium sensitivity (EC50) in both slow (**D**) and fast-twitch (**E**) myofibers. One symbol represents one patient. Significance calculated using paired t test or Wilcoxon test, depending on distribution of the data

Contractility of single myofibers over time are shown in Fig. 2. Figure 2A shows an example of a myofiber mounted in the experimental setup for contractility assays and (top right) schematic overview of the setup that was used for the contractility measurements. The number of myofibers assessed in each patient was 9 ± 4 slow-twitch myofibers and 12 ± 4 fast-twitch myofibers per biopsy. Maximum force decreased significantly in both slow- (*p* = 0.035) and fast-twitch myofibers (*p* = 0.048) (Fig. 2B, E). There were no significant changes in maximum normalized force or calcium sensitivity of force.

### Comparisons between in vitro muscle strength in critically ill patients and healthy controls

**Fig. 3:**
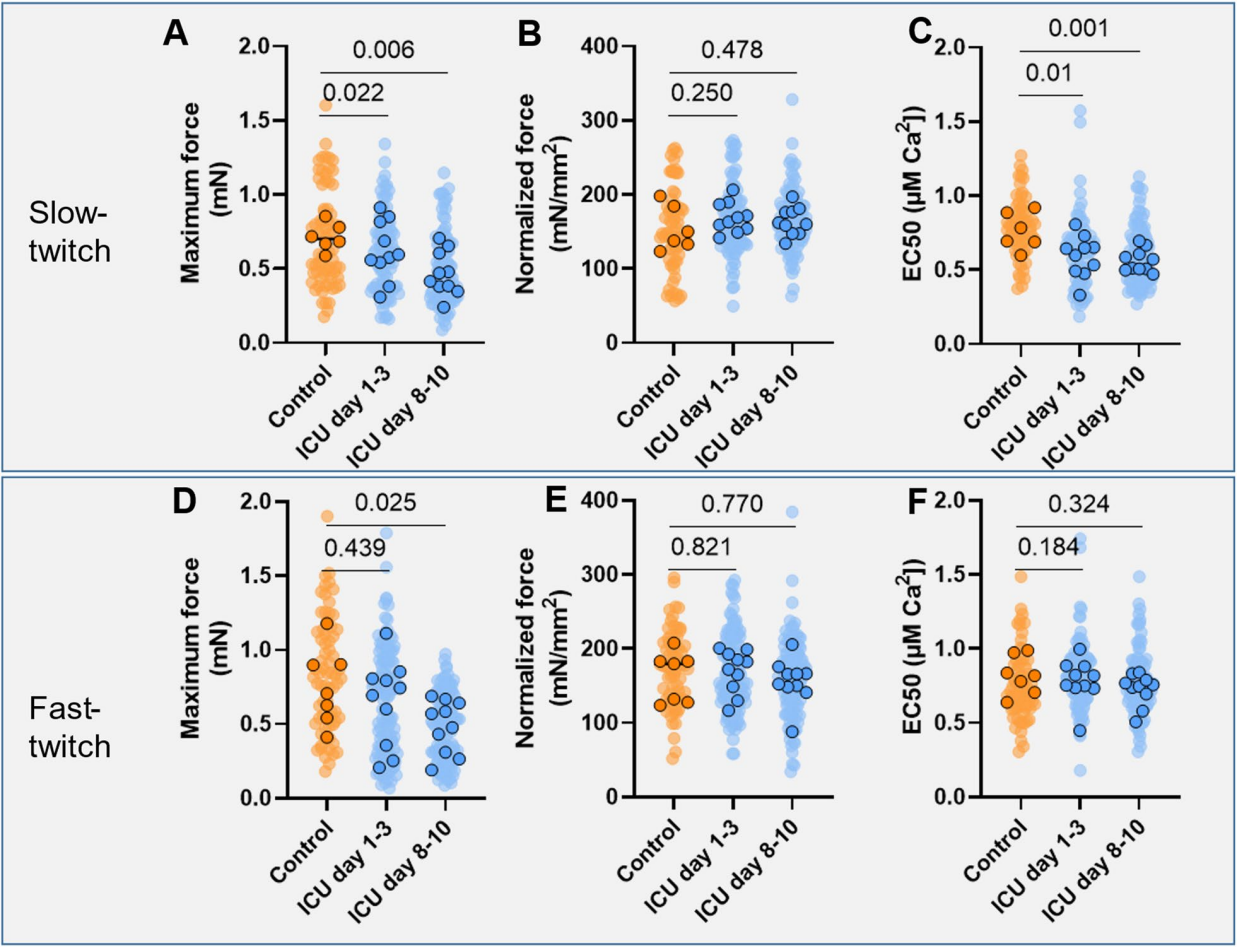
Contractility of single myofibers, comparisons between critically ill patients and healthy controls. Maximum force (**A**, **D**), normalized force (**B**, **E**) and calcium sensitivity (**C**, **F**) in slow- (top) and fast-twitch (bottom) myofibers in both the control and intervention group. Symbols in front represents patients, symbols in the back represent single myofibers. Significance calculated using linear mixed models

Comparisons between in vitro muscle strength in healthy controls and ICU patients are presented in Fig. 3. Compared to healthy controls, maximum force was significantly lower at ICU day 1–3 in the slow-twitch fibers (*p* = 0.022), and day 8–10 in both the slow-twitch (*p* = 0.006) and fast-twitch fibers (*p* = 0.025). Calcium sensitivity of force of the slow-twitch fibers was significantly lower at both ICU day 1–3 (*p* = 0.011) and day 8–10 (*p* = 0.001) compared to the healthy controls.

### In vivo measurements in critically ill patients

**Fig. 4:**
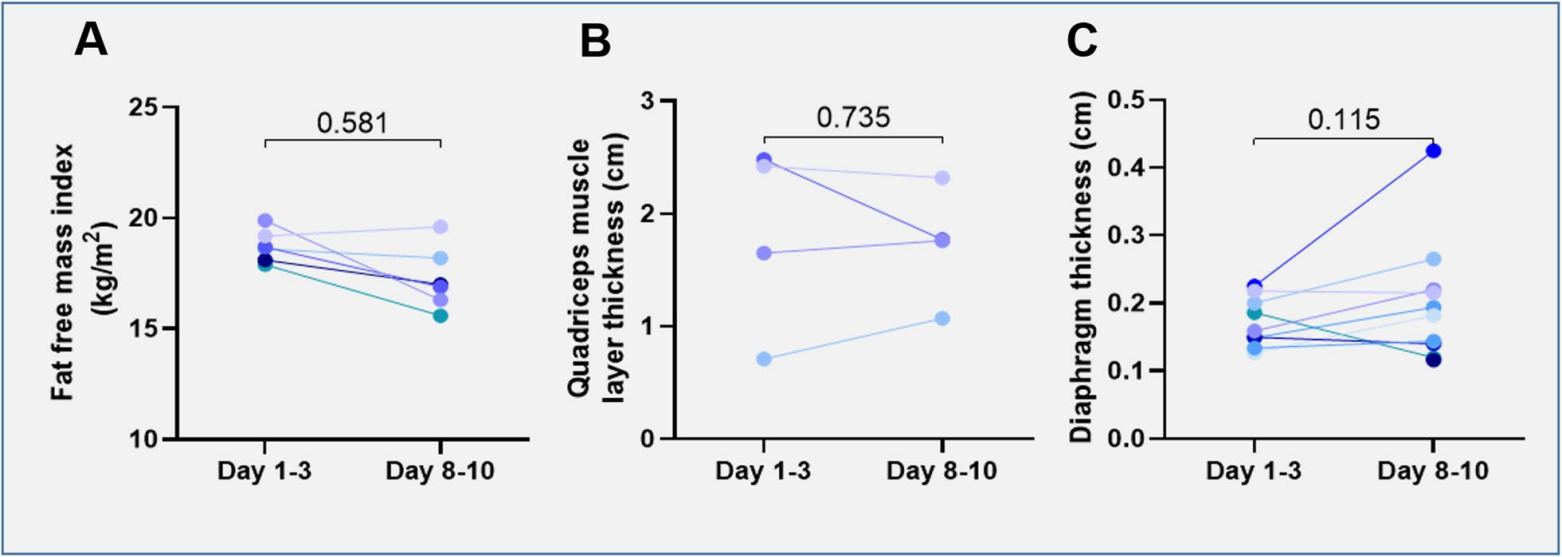
Fat-free mass index, quadriceps muscle layer thickness and diaphragm thickness over time and comparison of changes between groups. Changes in **A** fat-free mass index, **B** quadriceps muscle layer thickness **C** and diaphragm thickness **C** over time. One symbol represents one patient

FFMI did not change over time (*p* = 0.058, Fig. 4A). QMLT and diaphragm thickness also did not change over time (*p* = 1.000 and *p* = 0.192, respectively, Fig. 4B, C).

### Correlations

**Fig. 5:**
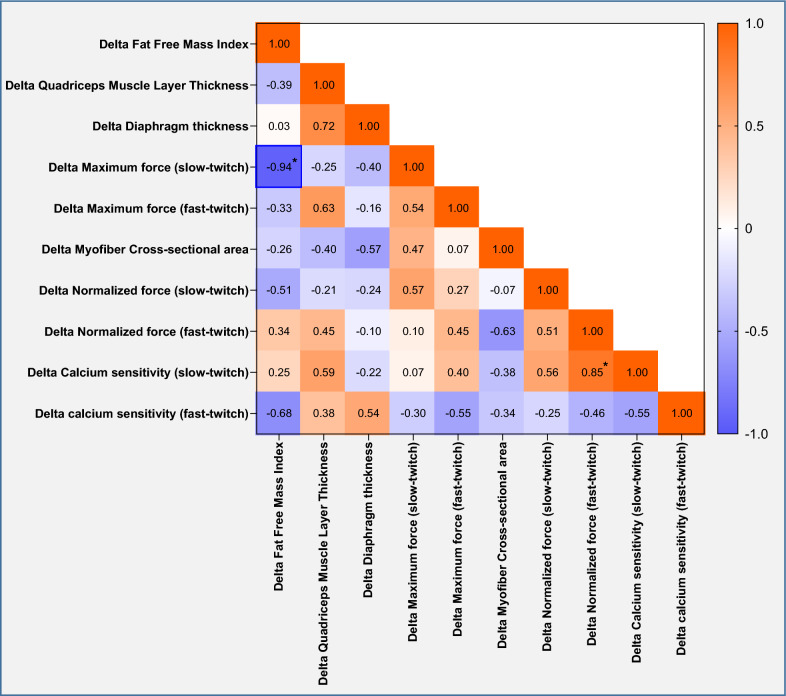
Matrix of correlations between changes in in vitro and in vivo measurements over time. Correlation matrix showing the Pearson *r* and Spearman statistics. Significant correlations are highlighted with a blue box

**Fig. 6:**
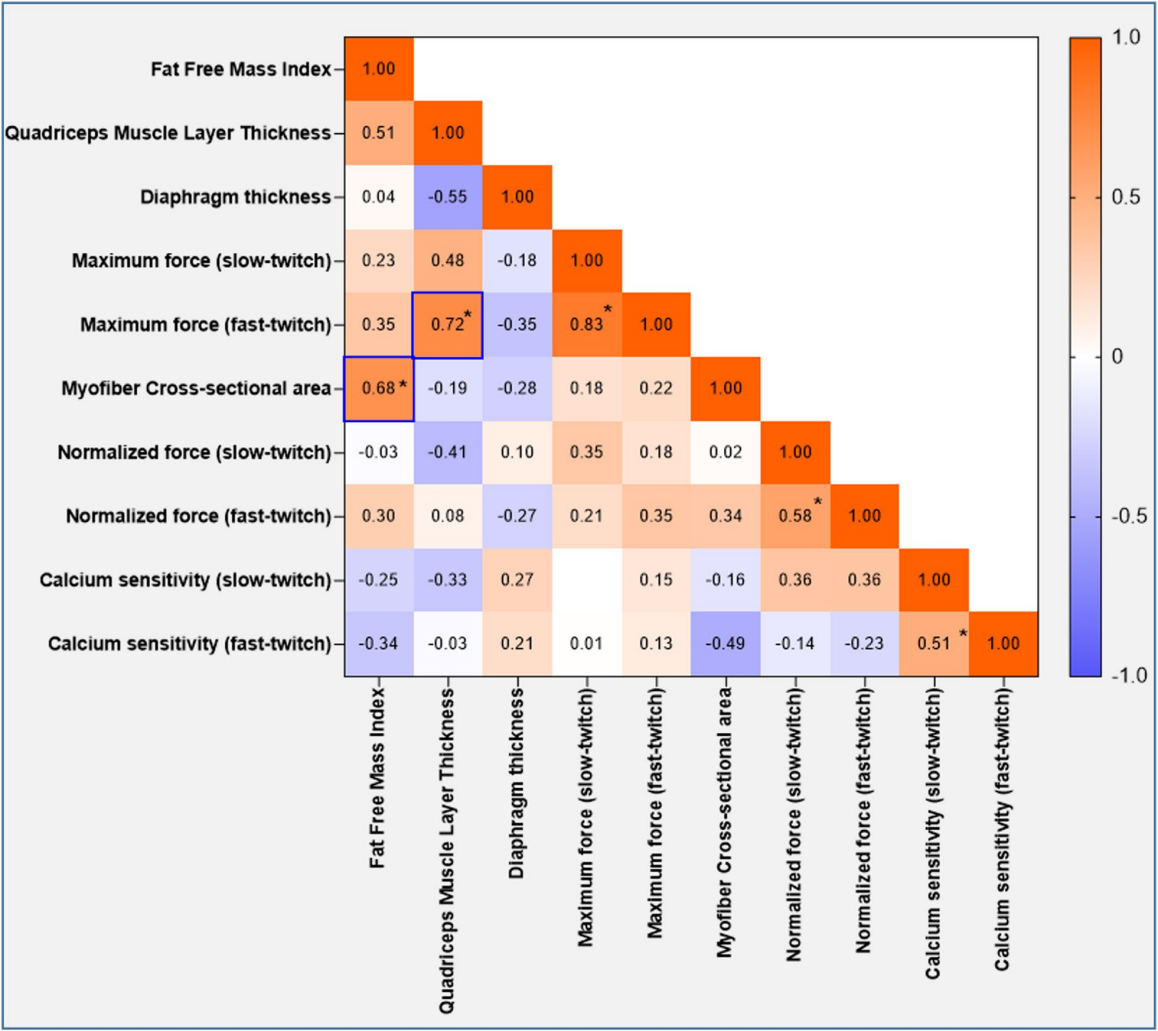
Matrix of correlations between changes in in vitro and in vivo measurements. Correlation matrix showing the Pearson *r* and Spearman statistics. Significant correlations are highlighted with a blue box

**Fig. 7:**
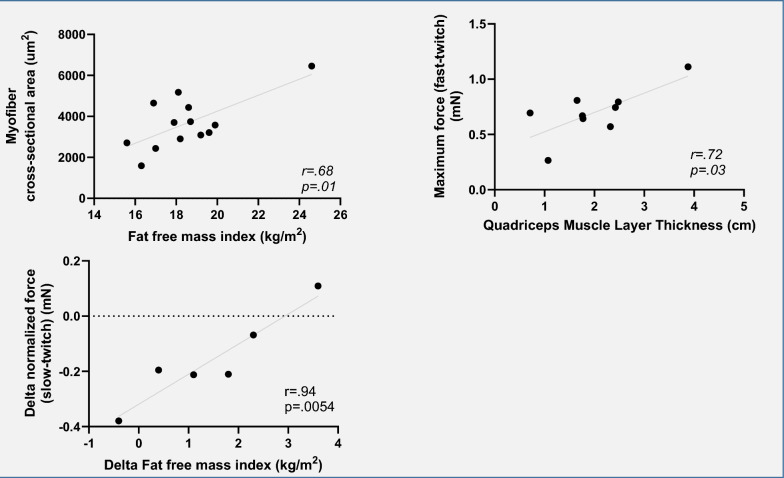
Scatter plots. Showing the correlation between myofiber cross-sectional area vs. fat-free mass index (left), maximum force of fast-twitch fibers vs. quadriceps muscle layer thickness (right) and change in fat-free mass index over time vs. change in normalized force of slow-twitch fibers over time

First, we studied correlations between in vitro and in vivo measurements expressed as change over time. There was a significant negative correlation between change in FFMI vs. change in maximum contractile force of myofibers (*r* = − 0.94, *p* = 0.005) and a significant positive correlation of change in calcium sensitivity of slow myofibers vs. normalized force of fast myofibers (*r* = 0.85, *p* = 0.002). For the separate measurements, we found a moderate correlation between myofiber CSA vs. FFMI (*r* = 0.68, *p* = 0.010) and between maximum force of the fast-twitch fibers vs. QMLT (*r* = 0.72, *p* = 0.029). Correlation matrices are presented in Figs. 5 and 6. Scatter plots for the significant correlations between bedside measurements and myofiber contractility are shown in Fig. 7. Correlation matrices for corresponding p-values are presented in Supplementary File, Figure S1 and S2.

## Discussion

Fiber maximum force declined significantly during the first week of critical illness. Furthermore, maximum force after 1 week of ICU stay was lower compared to healthy controls in both fiber types. At day 1–3 after ICU admission, maximum force was lower in slow-twitch myofibers compared to healthy controls. Both at the beginning of ICU stay and after 1 week of ICU stay, calcium sensitivity of slow-twitch fibers was lower compared to healthy controls. Measurements of myofiber CSA were moderately correlated with FFMI. Furthermore, measurements of maximum force of the fast-twitch fibers were moderately correlated with QMLT. However, the sample size available for these correlations was small.

We did not find significant changes in in vitro nor in vivo muscle mass. The absence of a significant reduction in myofiber CSA between day 1–3 and day 8–10 after ICU admission is not in line with the literature. In a landmark study investigating cross-sectional atrophy in 28 ICU patients, quadriceps myofiber CSA was reduced by 17.5% after 7 days [18]. In our study, mean myofiber CSA also declined with 17% after 7 days, but this difference was not statistically significant, probably due to the lower number of patients included in our study. Furthermore, our data originate from a RCT where all patients received standardized exercise, which may have attenuated muscle loss.

In a study assessing myofiber CSA and contractility of single myofibers isolated from sequential tibialis anterior biopsies in neuro-ICU patients, significant atrophy and loss in normalized force generation capacity was observed, but the authors did not differentiate between fiber types [14]. This discrepancy with our data may be explained by a distinct susceptibility to muscle wasting between the tibialis anterior, a predominantly fast-twitch muscle, with the quadriceps femoris, a muscle with approximately 40% slow-twitch myofibers [19, 20]. Furthermore, in contrast with the patients in the aforementioned study, the patients included in this study received a standardized exercise regimen, to avoid full unloading of the quadriceps. Thus, the absence of changes in normalized force or calcium sensitivity in our data may be due to not fully unloading the muscle. In the diaphragm of mechanically ventilated ICU patients, unloading by the ventilator has been shown to reduce normalized force and calcium sensitivity due to an increase in the population of myosin heads that is stuck in the super-relaxed state (SRX) [21]. In our study, calcium sensitivity and normalized force did not decrease during the first week of critical illness. Therefore, it is unlikely that an increase in SRX myosin, or dysfunction of sarcomeres in general, play a role in the early development of ICU-acquired weakness. Even though we did not observe a significant decrease in myofiber CSA in muscle cross-sections, maximum force of isolated myofibers decreased. In the absence of a decrease of normalized force or calcium sensitivity, decreased maximum force may be explained by a decreased CSA of single myofibers that were used for force measurements, even if a significantly decreased CSA was not observed in the muscle cross-sections.

To account for possible changes in muscle contractility before day 1–3 after ICU admission, we compared the data from ICU patients to those of healthy controls. Maximum force of slow-twitch myofibers was significantly lower at both time points, while the maximum force of fast-twitch myofibers was only lower at day 8–10. The absence of a significant difference in maximum force of fast-twitch myofibers at day 1–3 indicates that the changes happen later in the fast-twitch myofibers. Calcium sensitivity of slow-twitch myofibers at day 1–3 and day 8–10 after ICU admission was significantly higher compared to healthy controls. Low pH and changes in sarcomere length are known to increase calcium sensitivity, but are tightly controlled during our contractile experiments, and are, therefore, unlikely to contribute to our findings [22]. Another explanation for increased calcium sensitivity may be a decreased population of SRX myosin. Future research should investigate the exact underlying mechanism of these changes.

We sought to determine how QMLT would relate to myofiber contractility. QMLT was assessed with minimal pressure on the ultrasound probe, as suggested by a recent review because maximal pressure may alter myofascial structures [8]. Based on the observations in this study, QMLT may be a surrogate marker of muscle strength and ICU-acquired weakness. Interestingly, we observed a moderate correlation between maximum force of fast-twitch myofibers and QMLT in the absence of significant correlations with maximum force of slow-twitch fibers. This may be due to fast-twitch myofibers making up a larger part of QMLT compared to slow-twitch myofibers. Indeed, in our data, fast-twitch myofibers were more numerous. Furthermore, fast-twitch myofibers have a larger CSA in other studies performed on quadriceps tissue [23, 24]. No correlations were found between QMLT and any other measurements. We did not find a correlation between QMLT and myofiber CSA. This may be due to a lack of statistical power or other factors affecting the measurements. First, there may have been considerable inter-observer variability as no interrater or intra-rater reproducibility was assessed. Second, fluid status was not considered, but may have impacted the measurements. Finally, replacement fibrosis, a process where contractile protein is replaced with connective tissue, leading to unaltered muscle thickness in the presence of myofiber atrophy, may play a role [25]. In accordance with our findings, ultrasound muscle thickness measurements showed good discriminatory power to detect muscle weakness in a recent study in conscious ICU patients [26]. In conclusion, QMLT measurements appear to be a marker for muscle strength, but given the small sample size available for these correlations, further evaluation is needed.

Contrasting findings have been reported regarding the association of BIA-derived FFM with other body composition measurement modalities. Despite strong correlation, absolute muscle mass values of BIA vs. CT differed [27, 28]. Both hydration status of the patient and use of different equations in both BIA and CT methods might play a role, as they influence results. Nonetheless, low muscle mass as measured by BIA has previously been shown to correspond to low CT-derived muscle mass in ICU patients [28, 29]. In the current study, we found a moderate correlation between myofiber CSA and FFMI, indicating agreement between BIA derived FFM and in vitro measured quadriceps muscle mass. However, the sample size for these correlations was small. There was a significant negative correlation between the change in FFMI and change in absolute force of slow-twitch myofibers. This unexpected finding may be explained by the low number of patients that had BIA measured at the two time points (*n* = 5), making a chance finding more likely.

Our study has several limitations. First, our small study population is a clear limitation. Especially considering the in vivo measurements, very limited data was available. Also, in our cohort of ICU patients, only a single patient had sepsis, which has been shown to be a major driver of muscle atrophy [30]. This limits the generalizability of our data to the general ICU population. Second, we only analyzed data of patients who had a second biopsy taken at day 8–10, because we aimed to assess the change of myofiber contractility over time. As patients who died before the second biopsy were hereby excluded, we cannot rule out survivorship bias within our study. Third, changes in myofiber contractility over time were highly heterogeneous, possibly reflecting the heterogeneous patient population. Future studies investigating ways to monitor muscle mass and strength in ICU patients should be adequately powered, and patients should be carefully selected based on clinical phenotype. Finally, most patients were admitted to the ICU with traumatic brain injury, limiting generalization of the results.

## Conclusions

To conclude, maximum force of myofibers declined during the first week of critical illness, while there was no decrease of maximum normalized force or calcium sensitivity. Additionally, maximum force of myofibers was lower in the ICU group compared to healthy controls. Therefore, loss of contractile material and not dysfunction of sarcomeres likely drives muscle weakness in this cohort of patients.

## Supplementary Information

Supplementary Material 1.

## Data Availability

The datasets used and/or analyzed during the current study are available from the corresponding author on reasonable request.

## Abbreviations

BIA: Bio-electrical impedance
BMI: Body mass index
CSA: Cross-sectional area
CT: Computed tomography
ICU: Intensive care unit
ICU-AW: Intensive care unit-acquired weakness
FFM: Fat-free mass
FFMI: Fat-free mass index
RCT: Randomized controlled trial
SOFA: Sequential organ failure assessment
QMLT: Quadriceps muscle layer thickness
