# Supplementary material for: In vitro and in vivo muscle mass and strength during the first week of critical illness
Source: Intensive Care Med Exp. 2025 Jun 3;13:57. doi: 10.1186/s40635-025-00755-7 (PMC12133653; doi:10.1186/s40635-025-00755-7)
Supplement: Supplementary file 1 — Supplementary Material 1. [file 40635_2025_755_MOESM1_ESM.docx]

**Supplementary File**

***Original study interventions and methods***

Intervention procedures

Both the control and intervention group received a standardized exercise training program twice a day, starting on the day of inclusion and continuing throughout the ICU stay. Exercise training started with passive motion therapy and passive cycling for 30 min/day if tolerated by the patient. Resistance during cycling was gradually increased. A trained physiotherapist performed the second exercise session by passive stretching of the extremities according to a strict protocol. If patients were cooperative, active exercise and resistance training was started and gradually intensified according to the protocol in Table S2.

Both groups received standard enteral nutrition with a protein provision of 1.0 g/kg/day. The intervention group received an additional whey protein supplement (Beneprotein; Nestlé), distributed over four portions throughout the day, to increase total protein provision to 1.5 g/kg/day. Protein targets were based on pre-admission body weight. Enteral nutrition was initiated within 24 hours after ICU admission with gradually increasing amounts aiming to reach at least 80% of energy and protein targets on day 4, and 100% at day 7. Study nutrition was continued until oral intake was resumed (and enteral nutrition was discontinued) or until ICU discharge. Energy requirements were based on VCO_2_ derived from the mechanical ventilator as it is more accurate compared to predictive equations [31]. Nutrition in both groups was isocaloric (corrected for calories from propofol) thus, additional calories derived from the whey protein supplement in the intervention group were compensated by providing fewer calories from other nutrients. Demographic and clinical data were extracted from the patient data management system (EPIC).

Randomization, blinding, and treatment allocation

After informed consent was obtained, computer-based block randomization was performed by a researcher of the study team. Patients were allocated to the intervention or control group. Researchers performing *in vitro* measurements were blinded for group allocation. Treating intensivists and researchers performing *in vivo* measurements were not blinded.

Outcomes

The primary outcomes were changes in skeletal muscle strength as measured by contractile force of single myofibers isolated from the biopsies between day 1-3 and day 8-10 after ICU admission: maximum force (mN), normalized force (mN/mm2) and calcium sensitivity (EC50, [Ca2+]) of both slow-twitch and fast-twitch myofibers.

Secondary outcomes were changes in muscle mass between day 1-3 and day 8-10 after ICU admission as measured *in vitro*: by myofiber cross-sectional area (CSA, um^2^) and percentage of fast-twitch fibers (%) from cryosections, and *in vivo*: fat free mass (FFM, kg) and fat free mass index (FFMI, kg/m2) as measured by BIA and quadriceps muscle layer thickness (QMLT, cm) and diaphragm thickness as measured by ultrasound.

Analysis of muscle biopsies

*Histology*

We used a previously described method for muscle specimen handling [1]. Cryosections (8 µm thick, perpendicular to myofiber direction) were cut from the frozen biopsies and stained to study 1) myofiber type using myosin heavy chain antibody (fast MHC, 1:1000 ab91506, Abcam), 2) myofiber cross sectional area using wheat germ agglutinin (WGA, 1:100 diluted in PBS-1% BSA, Molecular Probes) staining. Sections were imaged in the following week using a Keyence BZ-X800 Fluorescent widefield microscope with a 20X objective. Myofiber cross sectional area was obtained from whole cryosections using the MuscleJ-2 FIJI-plugin[2]. The proportion of Fast-twitch myofibers was determined by manually counting fibers.

*Myofiber contractility experiments*

For permeabilized myofiber contractility experiments, single myofibers were isolated from the biopsies and their contractile performance was determined as previously described[1]. Between collection and measurements, samples were stored in a freezer at -80⁰C. Myofibers were manually isolated in a relaxing solution at 5ºC. At both ends, two aluminum clips were attached. The myofibers were incubated for 10 minutes in cold (5°C) skinning solution to permeabilize the plasma membrane enabling activation of myofilaments with exogenous calcium. Myofibers were mounted between a model 403A force transducer (10 kHz) and a model 315C servo-motor (Aurora Scientific Inc, Ontario, Canada, step time 250 µs). Myofiber dimensions were measured by means of a camera device coupled to the objective. Myofiber length was determined with 100x magnification, depth and width were measured with 400x magnification (an elliptical cross-section of the myofiber was assumed). Injury was examined microscopically; in case of severe damage, loss of striation and other irregularities the myofibers were excluded. Myofibers were stretched to optimal length by setting sarcomere length at 2.5 μm with dedicated Aurora software. To ensure stable attachment of the myofiber in the clips, the myofiber was briefly maximally activated prior to the experiment, and when necessary, re-stretched to a sarcomere length of 2.5μm. Note that this brief activation was performed prior to the determination of myofiber dimensions. The myofiber was sequentially bathed in relaxing, pre-activation, and finally maximal activating solutions ([Ca^2+^] = 32µM), where the maximum force was measured (expressed as mN, or when normalized to myofiber cross sectional area as mN/mm^2^). To determine submaximal force generation, myofibers were sequentially bathed in solutions with [Ca^2+^] increasing from 0 to 32µM and the steady-state force was measured. The obtained force-[Ca^2+^] data were fitted to the Hill equation, providing the EC50 (i.e., the [Ca^2+^] producing a half-maximum force). Details of solutions composition are presented in Table 1. Only myofibers with reproducible force generation were included in the analyses, as indicated by <10% force rundown during the contraction protocol. Rundown was assessed by comparing the force at pCa 4.5 at the beginning of the protocol to that at the end of the protocol, after the active stiffness assay. After completion of the contractility studies, the myofibers were collected and myofiber type was determined by sodium dodecyl sulfate–polyacrylamide gel electrophoresis, as described previously[3].

| **Table S1. Composition of solutions** | |
| --- | --- |
| Solution | Composition |
| Relaxing solution | 100 mM BES, 14.5 mM creatine phosphate, 6.97 mM EGTA, 40.76 mM K-propionate, 6.48 mM MgCl_2_, 5.89 mM Na_2_-ATP, and low concentration of freshly added protease inhibitors |
| Skinning solution | Relaxing solution with 1% Triton X-100, protease inhibitors 1.0 mM DTT, 0.24 mM PMSF, 0.04 mM leupeptin, 0.01 mM E64 |
| Pre-activating solution | 100 mM BES, 14.5 mM creatine phosphate, 0.1 mM EGTA, 6.9 mM HDTA, 41.14 mM K-propionate, 6.42 mM MgCl_2,_ 5.87 mM Na_2_-ATP |
| Maximal activating solution (pCa 4.5) | 100 mM BES, 7.0 mM CaEGTA, 14.5 mM creatine phosphate, 46.64 mM K-propionate, 6.28 mM MgCl_2,_ 5.97 mM Na_2_-ATP |
| Submaximal activating solutions | By accurate mixing relaxing solution and maximal activating solution, submaximal activating solutions with pCa7, 6.2, 5.8, 5.6 and 5.4 were made |
| Relax-glycerol solution | 50% (v/v) glycerol and relaxing solution, protease inhibitors 1.0 mM DTT, 0.24 mM PMSF, 0.04 mM leupeptin, 0.01 mM E64 |
| Relax-glycerol solution, high  inhibitors | 50% (v/v) glycerol and relaxing solution, protease inhibitors 1.0 mM DTT, 0.24 mM PMSF, 0.4 mM leupeptin, 0.1 mM E64 |
| *All solutions had an ionic strength of 180 mM and PH 7.1 | |

| **Table S2.** Exercise protocol | | | | | | | |
| --- | --- | --- | --- | --- | --- | --- | --- |
| **Level** | | **0**  **Non**  **cooperative** | **1**  **Non/barely cooperative** | **2**  **Somewhat cooperative** | **3**  **Not fully cooperative** | **4**  **Fully cooperative** | **5**  **Fully cooperative** |
| **SQ5** |  | 0 | <3 | 3 | 4-5 | 5 | 5 |
| **Assessment** |  | Negative | Positive, neurological or surgical conditions hinder transfer to chair | Positive, neurological or surgical conditions hinder transfer to chair (even with MRC sum score 36) | Positive | Positive | Positive |
| **MRC sum score** |  |  |  |  | 36 | 48 | 48 |
| **Intervention**  **/transfer training** | Lateral position | Yes | Yes | Yes | Yes |  |  |
|  | Fowler’s | Yes, semi | Yes | Yes, high | Yes, high | Yes, high | Yes, high |
|  | Chair |  |  | Yes, passive transfer | Yes, passive transfer | Yes, active transfer | Yes, active transfer |
|  | Stand |  |  |  | Yes, with help of two people | Yes, with help of one person | Yes |
|  | Walk |  |  |  |  | Yes, with tools | Yes |
| **Exercise 1** | 20-30 min |  | Bed bike 20’ passive | Bed bike 20’ passive/active | Bed bike 20’ active | Bed bike 20’ active | Bed bike 20’ active |
| **Exercise 2** |  |  | Stretch or resistance | Resistance | Resistance | Resistance | Resistance |

***Outcomes***

| **Table S3.** Outcomes | | |
| --- | --- | --- |
|  | **ICU patients (n=10)** | **Healthy controls (n=10)** |
| **Weight (kg)  Day 1-3  Day 8-10  Change** | 82.4 ± 14.7 85.3 ± 19.7 2.9 ± 5.9 | 82.3 ± 19.8 |
| **Myofiber cross sectional area (um2)  Day 1-3  Day 8-10  Change** | 4319 ± 1037 3583 ± 1513 -737 ± 1254 |  |
| **Myofiber type (fast-twitch) (%)  Day 1-3  Day 8-10  Change** | 49 ± 11 56 ± 19 7 ± 23 |  |
| **Maximum force (slow-twitch) (mN)  Day 1-3  Day 8-10  Change** | 0.62 ± 0.20 0.47 ± 0.15 -0.16 ± 0.20 | 0.72 ± 0.09 |
| **Maximum force (fast-twitch) (mN)  Day 1-3  Day 8-10  Change** | 0.64 ± 0.29 0.48 ± 0.18 -0.16 ± 0.22 | 0.75 ± 0.26 |
| **Normalized force (slow-twitch) (mN/mm2)  Day 1-3  Day 8-10  Change** | 169.1 ± 20.1 164.2 ± 18.7 -4.9 ± 22.3 | 154.5 ± 30.6 |
| **Normalized force (fast-twitch) (mN/mm2)  Day 1-3  Day 8-10  Change** | 169.3 ± 29.0 156.0 ± 30.0 -13.3 ± 42.8 | 162.6 ± 33.7 |
| **Calcium sensitivity (slow-twitch)  (EC50 [Ca2+])  Day 1-3  Day 8-10  Change** | 0.59 ± 0.14 0.56 ± 0.08 -0.04 ± 0.18 | 0.76 ± 0.13 |
| **Calcium sensitivity (fast-twitch)  (EC50 [Ca2+])  Day 1-3  Day 8-10  Change** | 0.78 ± 0.14 0.73 ± 0.11 -0.06 ± 0.12 | 0.82 ± 0.13 |
| **Fat free mass (kg)  Day 1-3  Day 8-10  Change** | 59.1 ± 10.0 52.6 ± 9.4 -5.2 ± 4.9 |  |
| **Fat free mass index (kg/m2)  Day 1-3  Day 8-10  Change** | 19.1 ± 2.6 17.2 ± 1.7 -1.7 ± 1.5 |  |
| **Quadriceps muscle layer thickness (cm)  Day 1-3  Day 8-10  Change** | 1.82 ± 0.83 1.73 ± 0.51 -0.09 ± 0.46 |  |
| **Diaphragm thickness (cm)  Day 1-3  Day 8-10  Change** | 0.172 ± 0.036 0.202 ± 0.092 0.040 ± 0.074 |  |

| **Table S4.** Duration of mechanical ventilation at the time of biopsies | | |
| --- | --- | --- |
| **Patient** | **Day 1-3 (hours)** | **Day 8-10 (hours)** |
| **1** | **45.5** | **213.5** |
| **2** | **17** | **185** |
| **3** | **40** | **167** |
| **4** | **5** | **168** |
| **5** | **33.25** | **201.25** |
| **6** | **44.33** | **260.33** |
| **7** | **50.5** | **218.5** |
| **8** | **28.25** | **196.25** |
| **9** | **79** | **247** |
| **10** | **49** | **217** |
| **Table S5.** Study day at which the biopsy was taken | | |
| **Patient** | **Biopsy #1 (day)** | **Biopsy #2 (day)** |
| **1** | **1** | **8** |
| **2** | **2** | **9** |
| **3** | **1** | **8** |
| **4** | **1** | **8** |
| **5** | **1** | **9** |
| **6** | **2** | **10** |
| **7** | **1** | **8** |
| **8** | **2** | **9** |
| **9** | **2** | **9** |
| **10** | **2** | **8** |

| **Table S6.** Numbers of measured myofibers per biopsy | | | |
| --- | --- | --- | --- |
| **Biopsy #** | **Slow myofibers**  **(mechanics)** | **Fast myofibers(mechanics)** | **Number of myofbers (CSA)** |
| **1.1** | **7** | **9** | **-** |
| **1.2** | **9** | **11** | **-** |
| **2.1** | **19** | **5** | **137** |
| **2.2** | **18** | **2** | **207** |
| **3.1** | **9** | **15** | **342** |
| **3.2** | **4** | **17** | **199** |
| **4.1** | **11** | **11** | **833** |
| **4.2** | **6** | **15** | **212** |
| **5.1** | **7** | **10** | **106** |
| **5.2** | **12** | **8** | **111** |
| **6.1** | **8** | **7** | **86** |
| **6.2** | **12** | **7** | **118** |
| **7.1** | **8** | **10** | **169** |
| **7.2** | **9** | **11** | **434** |
| **8.1** | **5** | **15** | **202** |
| **8.2** | **8** | **12** | **340** |
| **9.1** | **19** | **5** | **137** |
| **9.2** | **18** | **2** | **207** |
| **10.1** | **6** | **16** | **176** |
| **10.2** | **9** | **17** | **235** |
| **Control 1** | **8** | **5** | **-** |
| **Control 2** | **13** | **7** | **-** |
| **Control 3** | **9** | **11** | **-** |
| **Control 4** | **12** | **8** | **-** |
| **Control 5** | **-** | **21** | **-** |
| **Control 6** | **10** | **7** | **-** |
| **Control 7** | **10** | **9** | **-** |

***Supplementary Figures***


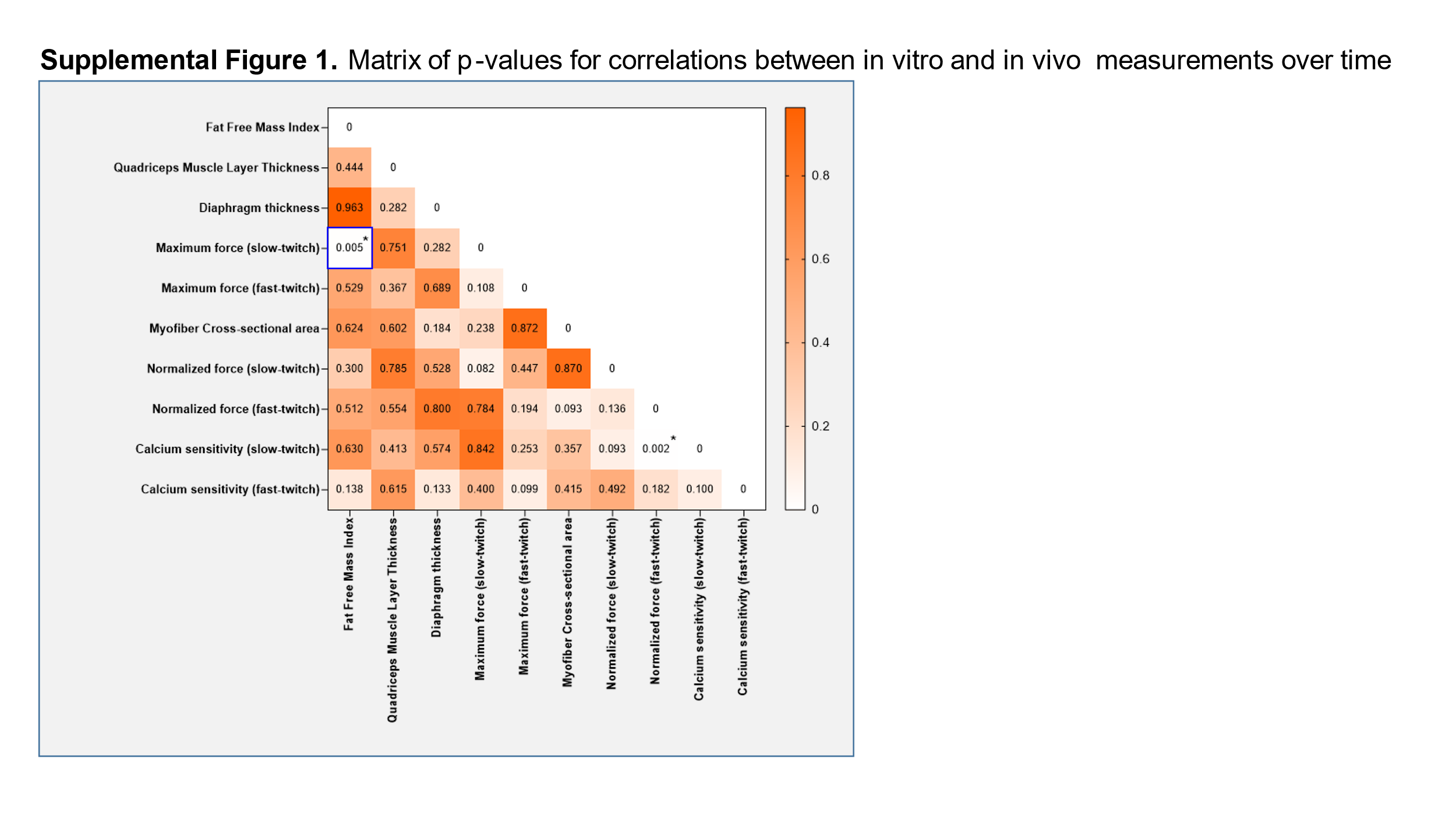


Figure S1. Matrix of p-values for correlations between *in vitro* and *in vivo* measurements over time

Correlation matrix showing the p-values for the correlations shown in Figure 5. Significant correlations between bedside and *in vitro* measurements are highlighted with a blue box.


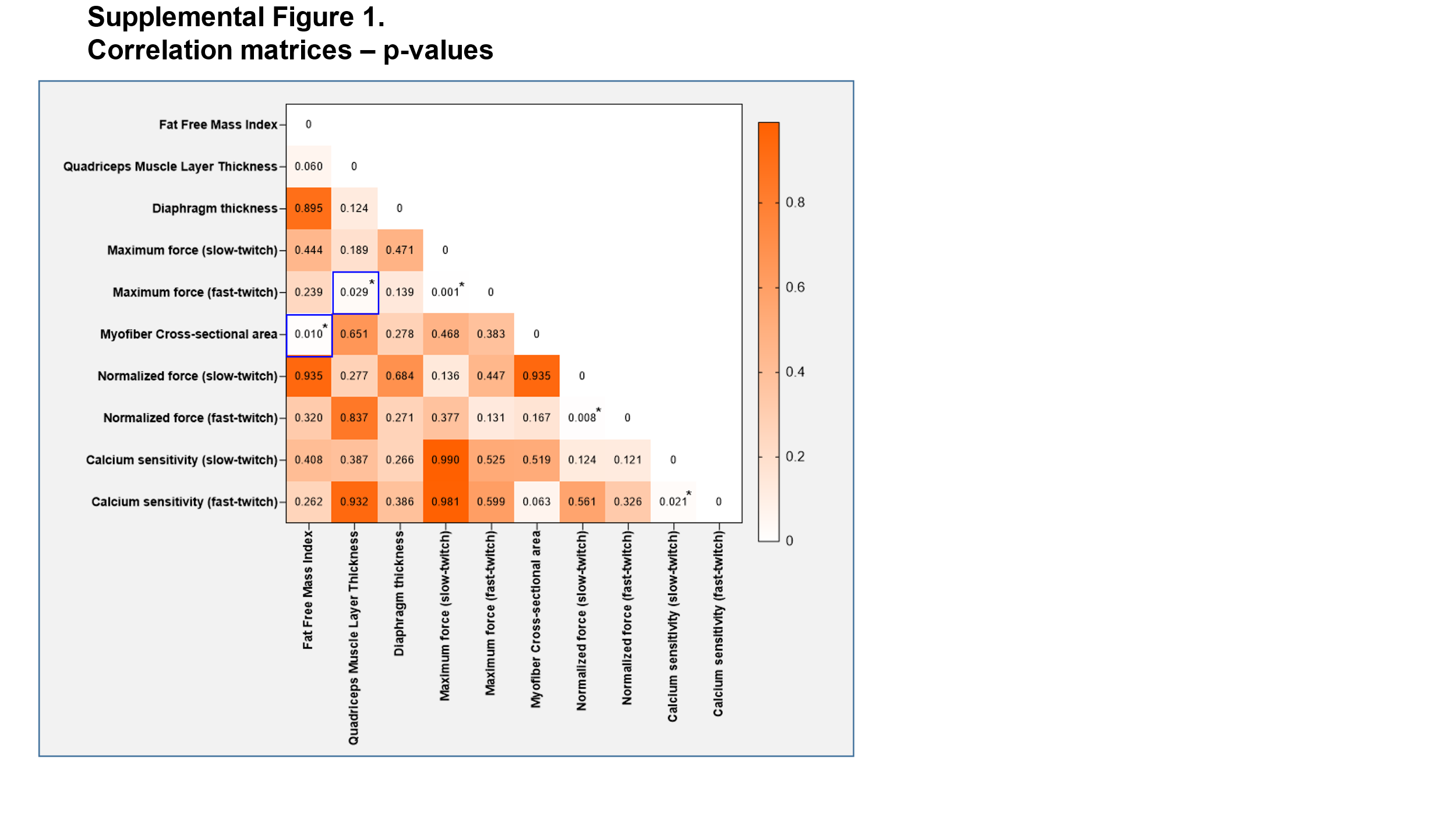
 Figure S2. Matrix of p-values for correlations between *in vitro* and *in vivo* measurements

Correlation matrix showing the p-values for the correlations shown in Figure 6. Significant correlations between bedside and *in vitro* measurements are highlighted with a blue box.

**References**

1. Ottenheijm CA, Heunks LM, Sieck GC, Zhan WZ, Jansen SM, Degens H, de Boo T, Dekhuijzen PN, (2005) Diaphragm dysfunction in chronic obstructive pulmonary disease. Am J Respir Crit Care Med 172: 200-205

2. Danckaert A, Trignol A, Le Loher G, Loubens S, Staels B, Duez H, Shorte SL, Mayeuf-Louchart A, (2023) MuscleJ2: a rebuilding of MuscleJ with new features for high-content analysis of skeletal muscle immunofluorescence slides. Skelet Muscle 13: 14

3. van den Berg M, Hooijman PE, Beishuizen A, de Waard MC, Paul MA, Hartemink KJ, van Hees HWH, Lawlor MW, Brocca L, Bottinelli R, Pellegrino MA, Stienen GJM, Heunks LMA, Wust RCI, Ottenheijm CAC, (2017) Diaphragm Atrophy and Weakness in the Absence of Mitochondrial Dysfunction in the Critically Ill. Am J Respir Crit Care Med 196: 1544-1558
